# ENOX2 (tNOX)–Associated Stemness in Oral Cancer Cells and Its Clinical Correlation in Head and Neck Tumors

**DOI:** 10.3390/antiox15010098

**Published:** 2026-01-13

**Authors:** Che-Wei Wang, Atikul Islam, Yu-Tung Shih, Chin-Fang Chang, Mu Kuan Chen, Pin Ju Chueh

**Affiliations:** 1 Graduate Institute of Biomedical Sciences, College of Medicine, National Chung Hsing University, Taichung 402202, Taiwan; d109059001@mail.nchu.edu.tw (C.-W.W.); islammiu555@gmail.com (A.I.); d109059004@mail.nchu.edu.tw (Y.-T.S.); 2 Department of Otorhinolaryngology-Head and Neck Surgery, Changhua Christian Hospital, Changhua 500209, Taiwan; 53780@cch.org.tw; 3 Department of Neurosurgery, Jen-Ai Hospital, Taichung 412224, Taiwan; 4 Department of Otolaryngology, Head and Neck Surgery, Jen-Ai Hospital, Taichung 412224, Taiwan; benglung@hotmail.com; 5 Graduate Institute of Chinese Medicine and Drug Development, College of Medicine, National Chung Hsing University, Taichung 402202, Taiwan

**Keywords:** cancer stem cells (CSCs), head and neck cancer, stemness, 3-D sphere culture, tumor-associated NADH oxidase (ENOX2; tNOX), sex-determining region Y box 2 (SOX2), silent information regulator 1 (Sirtuin1; SIRT1)

## Abstract

Cancer remains one of the most common causes of death worldwide and imposes enormous social and economic burdens. Human tumor-associated NADH oxidase (ENOX2, also known as tNOX) is a cancer cell-specialized NADH oxidase that is expressed on the membranes of cancer cells. In this study, we investigated the potential role of ENOX2 in regulating stemness properties in oral cancer through a combination of in vitro, in vivo, and bioinformatics approaches. We found that ENOX2 physically interacted with the stem cell transcription factor, SOX2, in co-immunoprecipitation experiments. The expression and activity of ENOX2 were elevated in *p53*-functional SAS and *p53*-mutated HSC-3 oral cancer cell spheroids compared with their monolayer counterparts. Consistently, SIRT1, a downstream effector modulated by ENOX2 through NAD^+^ generation, was also upregulated in spheroid cultures. Functional studies further established that ENOX2 overexpression significantly enhanced spheroid formation, self-renewal properties, stem cell marker expression, and PKCδ expression, whereas ENOX2 knockdown produced the opposite effects. In xenograft models, ENOX2-overexpressing oral cancer cell spheroids exhibited enhanced tumorigenicity, while ENOX2-silenced spheroids formed significantly smaller tumors. Complementary analyses of public transcriptomic and proteomic datasets revealed elevated ENOX2 expression in human head and neck tumor tissues compared with adjacent normal tissues. Based on these findings and literature-supported correlations, we propose a putative ENOX2-SIRT1-SOX2 regulatory framework that may contribute to the acquisition and maintenance of stem-like properties of oral cancer cells. While the ENOX2–SOX2 interaction was experimentally validated, the roles of SIRT1 and other downstream components are inferred from bioinformatic analyses and prior studies; thus, this axis represents a hypothetical model that warrants further mechanistic investigation. Collectively, our results identify ENOX2 as a potential regulator of oral cancer stemness and provide a conceptual foundation for future studies aimed at elucidating its downstream pathways and clinical relevance in head and neck tumors.

## 1. Introduction

According to GLOBOCAN 2022 estimates, lip and oral cavity cancers accounted for approximately 389,846 new cases and 188,438 deaths worldwide, ranking as the 15th leading cause of cancer-related mortality [1]. The predominant histological subtype is oral squamous cell carcinoma (OSCC), which arises from the epithelial lining of the oral cavity, pharynx, and larynx, and represents nearly 90% of all oral malignancies. Despite advances in surgery, radiotherapy, and chemotherapy, no substantial improvement in 5-year survival rates has been observed in recent years [2], largely due to local recurrence, metastasis, and therapeutic resistance. To address this challenge, Taiwan has implemented one of the most comprehensive nationwide oral cancer screening programs, focusing on high-risk individuals such as habitual betel-nut chewers, smokers, and alcohol users. Nevertheless, tumor recurrence and metastasis remain persistent challenges despite these large-scale preventive efforts [3,4]. These clinical realities highlight the urgent need for novel therapeutic strategies that can effectively prevent relapse and improve long-term outcomes.

OSCC typically arises from the mucosal epithelium of the tongue, buccal mucosa, gingiva, and floor of the mouth. Clinically, it often presents as a persistent ulcer or exophytic lesion and is commonly diagnosed at an advanced stage due to the asymptomatic nature of early disease [5]. Histopathologically, OSCC is characterized by epithelial dysplasia, keratin pearl formation, and stromal invasion. Current management strategies rely on surgical resection with or without neck dissection, followed by adjuvant radiotherapy or concurrent chemoradiotherapy for locally advanced disease [6,7]. Despite these multimodal interventions, the 5-year survival rate for patients with stage III–IV OSCC remains below 50%, and prognosis is strongly influenced by tumor stage, nodal metastasis, perineural invasion, and molecular alterations [8,9]. These clinicopathological challenges underscore the need to identify novel molecular regulators of OSCC progression and treatment resistance.

Stem cells possess unique abilities to self-renew and differentiate into various cell types, maintaining tissue homeostasis during normal development and repair. These processes are tightly regulated by transcriptional networks involving factors such as SOX2, Oct4, and Nanog, which sustain pluripotency and stem cell identity. In cancer, dysregulation of these mechanisms gives rise to cancer stem cells (CSCs), a subpopulation with enhanced self-renewal capacity, tumor-initiating potential, and resistance to conventional therapies. CSCs were first demonstrated in acute myeloid leukemia (AML), where a distinct subpopulation of cells was found to be capable of initiating leukemia in immunodeficient mice [10,11,12]. Subsequent studies revealed similar “stem cell-like” subpopulations in solid tumors, including breast, colorectal, and brain cancers [13,14,15,16,17]. CSCs express characteristic markers such as CD44, ALDH1, SOX2, Oct4, and Nanog, which are commonly used to identify and characterize stem-like populations in epithelial cancers. These cells are now recognized as key drivers of tumor initiation, maintenance, recurrence, and metastasis [18,19,20]. Their identification provides critical insights into therapeutic resistance and the mechanisms underlying tumor progression [19,21,22].

Because CSCs contribute to tumor heterogeneity and therapy resistance through their ability to self-renew and differentiate within a tumor, it is essential to study them in physiologically relevant contexts. Conventional two-dimensional (2D) monolayer cultures on polystyrene surfaces provide a simplified model that is easy to use but fails to capture the complex cell–cell interactions and microenvironment of tumors in vivo [23,24]. By contrast, three-dimensional (3D) culture systems, such as spheroid models, allow cancer cells to proliferate in all directions and form extracellular matrix components and cell junctions that better recapitulate in vivo conditions. This is particularly important for CSC research, as 3D models enable more accurate investigations of tumorigenesis, growth, metastasis, and recurrence [24,25,26,27,28]. Thus, 3D cell culture systems offer powerful tools for identifying CSCs and elucidating the mechanisms that drive tumor biology.

**Figure 1:**
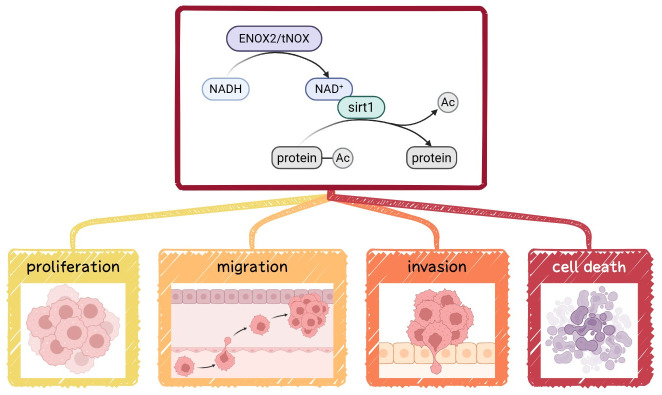
Conceptual model of ENOX2-SIRT1-mediated regulation of cancer cell functions. ENOX2/tNOX catalyzes the oxidation of NADH to NAD^+^, leading to activation of the NAD^+^-dependent deacetylase SIRT1. Activated SIRT1 deacetylates downstream proteins and transcription factors, influencing cancer cell proliferation, migration, invasion, and cell death.

ENOX2, also known as tumor-associated NADH oxidase (tNOX) or COVA1, is a cancer cell surface protein with both NADH oxidase and protein disulfide–thiol exchange activities. It has been implicated in promoting proliferation, growth, migration, and invasion across multiple cancer types [29,30,31,32,33]. By catalyzing the oxidation of NADH to NAD^+^, ENOX2 influences the activity of SIRT1, a member of the NAD^+^-dependent sirtuin family of deacetylases (Figure 1). SIRT1 critically regulates key cellular processes, including metabolism, differentiation, aging, and stem cell maintenance [34,35,36,37]. In this context, SIRT1 acts through the deacetylation of transcription factors, such as Oct4, Nanog, and SOX2, to balance self-renewal and differentiation and thereby sustain pluripotency [38,39,40,41]. Given these essential functions, the potential involvement of the ENOX2-SIRT1 axis in regulating stemness warrants deeper investigation.

In this study, we focused on oral cancer as a clinically urgent and regionally relevant disease model. Using 3D spheroid cultures, we examined the role of ENOX2 in maintaining stem-like properties in oral cancer cells. We further evaluated the impact of ENOX2 overexpression or knockdown on CSC characteristics in vitro and in vivo. Our results provide new insights into the therapeutic potential of targeting ENOX2 in CSC-directed cancer treatments.

## 2. Materials and Methods

### 2.1. Materials

The following antibodies were purchased from Cell Signaling Technology, Inc. (Danvers, MA, USA): anti-SIRT1 (1:1000, Cat#2496), anti-Nanog (1:2000, Cat#4903), anti-SOX2 (1:1000, Cat#2748), anti-Oct4 (1:1000, Cat#2750), anti-ABCG2 (1:1000, Cat#4477), anti-ALDH1 (1:1000, Cat#54135), anti-PKCδ (1:1000, Cat#2058), anti-c-Myc (1:1000, Cat#5605), and anti-GST (1:1000, Cat#2624). Rabbit anti-human CD133 (prominin-1) (1:50, Cat#ZRB1013) and mouse anti-rabbit IgG-FITC (1:200, Cat#AP160F) antibodies were obtained from Sigma-Aldrich (Burlington, MA, USA). Mouse anti-human CD44-FITC (1:50, Cat#11-0441-82) was sourced from Invitrogen (Carlsbad, CA, USA). The anti-β-actin antibody (1:10,000, Cat#60008-1-Ig) was from Proteintech Group, Inc. (Rosemont, IL, USA). The anti-mouse (1:20,000, Cat#115-035-003) and anti-rabbit IgG (1:20,000, Cat#111-035-003) secondary antibodies were purchased from Jackson ImmunoResearch Laboratories (West Grove, PA, USA). The antisera to ENOX2 (dilution 1:1000) used for immunoblotting were generated as previously described [42]. Capsaicin (Cat#M2028) and other chemicals were obtained from Sigma-Aldrich (Burlington, MA, USA). All antibodies and reagents used in this study are listed in Supplementary Table S1, including vendor, catalog number, and working dilution.

### 2.2. Cell Culture and Transfection

SAS (RRID:CVCL_1675; human squamous cell carcinoma of the tongue) and HSC-3 (RRID:CVCL_1288; human tongue squamous cell carcinoma) cells were kindly provided by Dr. Yuen-Chun Li (Department of Biomedical Sciences, Chung Shan Medical University, Taiwan). Cells were cultured in Dulbecco’s Modified Eagle Medium (DMEM) supplemented with 10% fetal bovine serum (FBS), 100 U/mL penicillin, and 50 µg/mL streptomycin (all from Thermo Fisher Scientific, Waltham, MA, USA). Cultures were maintained at 37 °C in a humidified incubator with 5% CO_2_, and the medium was replaced every 2–3 days.

Cells were transiently transfected with GST-ENOX2 or GST (control) using the jetPEI transfection reagent according to the manufacturer’s protocol (Polyplus-transfection SA, Illkirch Cedex, France). The full-length ENOX2(tNOX) cDNA was cloned into the pCMV–GST or pGEX-4T-1 GST expression vector using the BamHI and SalI restriction sites. For gene silencing, ON-TARGETplus ENOX2 siRNA (Cat#sc-91254) and non-targeting control siRNA (Cat#sc-37007) were purchased from Santa Cruz Biotechnology (Dallas, TX, USA). Cells were seeded in 10 cm dishes, allowed to attach overnight, and then transfected with ENOX2 siRNA or control siRNA using Lipofectamine RNAiMAX Reagent (Invitrogen, Thermo Fisher Scientific, Waltham, MA, USA) according to the manufacturer’s instructions.

### 2.3. Bioinformatics Analysis

Publicly available online databases were used to analyze the expression patterns, correlation, and prognostic significance of ENOX2, SIRT1, and SOX2. The UALCAN database (https://ualcan.path.uab.edu) was used to evaluate *ENOX2* and *SOX2* mRNA expression levels, as well as ENOX2 protein expression in head and neck cancers [43,44]. The Human Protein Atlas (HPA) (https://www.proteinatlas.org) was used to examine *ENOX2* mRNA and protein levels across various head and neck cancer cell lines. Overall survival and gene correlation analyses were performed using the Kaplan–Meier Plotter (https://www.kmplot.com/), while cBioPortal for Cancer Genomics (https://www.cbioportal.org/results/coexpression (accessed on 21 July 2024)) was used to access co-expression relationships among *ENOX2*, *SIRT1*, and *SOX2* [45,46]. In addition, the Integrated Interaction Database (IID) (https://iid.ophid.utoronto.ca/search_by_proteins/ (accessed on 29 July 2024)) was used to predict potential protein–protein interactions involving ENOX2 [47]. All analyses were conducted using default parameters unless otherwise specified.

### 2.4. Three-Dimensional Spheroid Formation Assay

When cell confluence reached around 80%, cells were detached using 1× trypsin–EDTA. After removal of trypsin-containing medium, the cells were suspended in DMEM supplemented with 20 ng/mL epidermal growth factor (EGF) and 20 ng/mL basic fibroblast growth factor (FGF2) (Trust Gene Biotech Ltd., Taipei, Taiwan). Cells were then seeded into ultra-low attachment 24-well plates (Corning, NY, USA) at a density of 1000 cells per well and incubated at 37 °C in a humidified atmosphere containing 5% CO_2_.

Poly(2-hydroxyethyl methacrylate) (polyHEMA) (Cat#25249-16-5; Sigma-Aldrich, MO, USA) was prepared at a concentration of 60 mg/mL in 95% ethanol at 65 °C for 2–3 h. Culture dishes were coated with the polyHEMA solution and dried under UV for 45 min. Cells were then plated in polyHEMA-coated 12-well plates (Corning, NY, USA) at a density of 2 × 10^5^ cells per well and incubated at 37 °C with 5% CO_2_.

### 2.5. Real-Time Cell Proliferation Monitoring Using the xCELLigence System

For continuous monitoring of cell proliferation, GST-vector and GST-ENOX2 overexpressed cells (1 × 10^4^ cells/well) were seeded into E-plates (Cat#6465412001, Agilent Technologies, Santa Clara, CA, USA). After incubation for 30 min at room temperature to allow cell attachment, the plates were transferred to the xCELLigence System (Roche, Mannheim, Germany). Cells were cultured for 4 days, and impedance was recorded every hour, as previously described. Cell impedance was expressed as the cell index (CI), calculated as CI = (Z_i_ − Z_0_) [Ohm]/15[Ohm], where Z_0_ represents background resistance and Z_i_ the resistance at a given time point. The normalized cell index was obtained by dividing the cell index at a given time point (CI_ti_) by that at the designated normalization time (CI_nml_time_).

### 2.6. Flow Cytometry Analysis of Cancer Stem Cell Surface Markers

Spherical cells obtained from the spheroid formation assay of SAS and HSC-3 cells were first verified for stem cell surface markers prior to subsequent experiments, to confirm enrichment of cancer stem cell populations after sphere culture. Tumor spheres were treated with 1:1 diluted 2.5% Trypsin-EDTA (Invitrogen, Carlsbad, CA, USA) for 5 min at 37 °C, washed, and dissociated by gentle pipetting. A total of 1 × 10^5^ cells were then stained with rabbit anti-human CD133 (prominin-1) and mouse anti-human CD44-FITC. CD133 is one of the most widely used markers for identifying and isolating cancer stem cell (CSC) populations across various tumors, including carcinomas. For CD133 staining, mouse anti-rabbit IgG-FITC was used as the secondary antibody. After three washes with PBS, cells were fixed in 1% paraformaldehyde (PFA) in PBS, and the fluorescence intensity was analyzed using a Beckman Coulter CytoFLEX LX flow cytometer (Brea, CA, USA) operated with CytExpert software, version 2.6.0 (Beckman Coulter, Brea, CA, USA).

### 2.7. Quantification of Intracellular NAD^+^/NADH Ratio

Intracellular levels of oxidized and reduced NAD were quantified using an NADH/NAD^+^ Quantification Kit (BioVision Inc., Milpitas, CA, USA) according to the manufacturer’s instructions. Briefly, 2 × 10^6^ cells were washed with cold PBS, pelleted, and extracted by two freeze–thaw cycles in 400 µL NADH/NAD^+^ extraction buffer. The extracts were vortexed and centrifuged at 14,000 rpm (18,407× *g*) for 5 min. Supernatants (200 µL) containing NADH/NAD^+^ were transferred to microcentrifuge tubes, heated at 60 °C for 30 min to decompose NAD^+^ while preserving NADH, and immediately placed on ice. Samples were centrifuged again, and the supernatants were transferred to a 96-well plate. NAD^+^ standards and cycling mix were prepared following the manufacturer’s protocol. A 100 µL aliquot of reaction mix was added to each well containing NADH standards or samples and incubated at room temperature for 5 min to convert NAD^+^ to NADH. Subsequently, the NADH developer solution was added, and the reaction was incubated at room temperature for 15–30 min. The reaction was then terminated with 10 µL Stop Solution, and absorbance was measured at 450 nm using a BioTek 800 TS Microplate Reader (Agilent Technologies, Santa Clara, CA, USA). The optical density (OD) at 450 nm was recorded at multiple time points between 1 and 30 min to monitor reaction kinetics.

### 2.8. Immunoprecipitation and Western Blot Analysis

For immunoprecipitation, protein extracts from cells grown in 100 mm dishes were incubated with 20 µL Protein G agarose beads (for rabbit antibodies) for 1 h at 4 °C with rotation to pre-clear nonspecific binding. GST, SOX2 antibody, or control IgG was then added to the beads in 500 µL lysis buffer (20 mM Tris-HCl, pH 7.4; 100 mM NaCl; 5 mM EDTA; 2 mM PMSF; 10 ng/mL leupeptin; 10 µg/mL aprotinin) and incubated overnight at 4 °C with rotation. The beads were collected by centrifugation at 3000 rpm for 2 min at 4 °C, and 80 µL of supernatant was reserved as input lysate. Beads were washed three times with lysis buffer, and bound proteins were eluted for subsequent Western blot analysis.

For Western blotting, cell lysates were prepared using the same lysis buffer. Equal amounts of protein (40 µg) were resolved by SDS–PAGE and transferred to PVDF membranes (Schleicher & Schuell, Keene, NH, USA). Membranes were blocked, washed, and incubated with the indicated primary antibodies overnight at 4 °C, followed by incubation with horseradish peroxidase-conjugated secondary antibodies for 1 h at room temperature. Protein bands were visualized using enhanced chemiluminescence (ECL) reagents (Amersham Biosciences, Piscataway, NJ, USA) and detected on X-ray film under a red safe light. Films were scanned, and band intensities were quantified using ImageJ software (version 1.8.0; NIH, Bethesda, MD, USA). Western blotting was performed using separate membranes for different target proteins. For each biological replicate, β-actin and two to three target proteins were analyzed on the same membrane and processed in parallel. Minor differences in molecular weight marker spacing may occur due to independent gel preparation and electrophoresis. Each β-actin blot served as the loading control for its corresponding targets. Western blot experiments for HSC-3 adherent and spheroid cells were not repeated, whereas repeated analyses were performed for SAS spheroids due to their consistent and robust induction of target proteins. For mouse tumor samples, tissue was available only from the 1 × 10^6^ cell injection group; tumors from lower-dose vector control groups were insufficient for protein extraction and were not analyzed. All Western blot data were normalized to β-actin and expressed as mean ± SD from three independent experiments. Statistical significance was evaluated using one-way ANOVA with Bonferroni’s post hoc test (*p* < 0.05). All analyses were performed on cell lysates only. The uncropped original Western blot images, quantitative analyses, and corresponding statistical data (bar graphs) are presented in the Supplementary Figures.

### 2.9. Xenograft Tumor Formation Assay in Mice

Specific pathogen-free (SPF) advanced severe immunodeficiency (ASID) mice were purchased from the National Laboratory Animal Center (Taipei, Taiwan). ASID mice lack functional T, B, and natural killer (NK) cells, providing a highly immunocompromised background that supports efficient engraftment and growth of human tumor xenografts. All animal experiments were conducted under protocols approved by the Institutional Animal Care and Use Committee of National Chung Hsing University (NCHU IACUC: 109-153). For the gain-of-function study, mice were randomized (with investigators blinded to allocation) into three groups. SAS cells transfected with either vector or GST-ENOX2 were subcutaneously inoculated into the right flank of mice with 1 × 10^6^ (*n* = 3 per group), 2 × 10^4^ (*n* = 5 per group), or 1 × 10^4^ (*n* = 3 per group) viable cells in 100 µL PBS. For the loss-of-function study, mice were divided into two groups and subcutaneously inoculated with 100 µL PBS containing 1 × 10^6^ SAS cells transfected with either siRNA-control (*n* = 3 per group) or siRNA-ENOX2 (*n* = 3 per group). Group sizes were determined a priori based on previous studies [48] and in accordance with the 3Rs principle, to minimize animal use while maintaining statistical validity. Tumor growth was monitored for 30 to 45 days post-inoculation. On the day of sacrifice, mice were anesthetized by intraperitoneal injection of Avertin (0.5 mg/g body weight) and blood was collected by cardiac puncture to ensure euthanasia. Tumor length (L) and width (W) were measured every two days, and tumor volume (V) was calculated as V = L × W^2^ × 0.5. Differences in tumor size were assessed using one-way ANOVA, with *p* < 0.05 considered statistically significant.

### 2.10. Capsaicin Treatments

Capsaicin (Sigma-Aldrich, Burlington, MA, USA; Cat#M2028) was dissolved in DMSO to prepare a 400 mM stock solution and stored at –20 °C. Working concentrations of 200 µM and 400 µM were prepared by 2000- and 1000-fold dilution of the stock solution, respectively, in the corresponding culture medium. Cells or spheroids were treated with capsaicin for 5 days, and control groups received an equivalent volume of DMSO (<0.1%).

### 2.11. Statistics

All data are expressed as mean SD from three independent experiments. Statistical analyses were performed using SigmaPlot 12.5 (Systat Software Inc., San Jose, CA, USA) or IBM SPSS Statistics version 20 (IBM Corp., Armonk, NY, USA). Differences between control and treatment groups were assessed using one-way ANOVA, followed by Bonferroni’s post hoc test for pairwise comparisons. A value of *p* < 0.05 was considered statistically significant.

## 3. Results

### 3.1. Expression Levels of ENOX2, SIRT1, and SOX2 Are Upregulated in Patients with Oral Cancer

**Figure 2:**
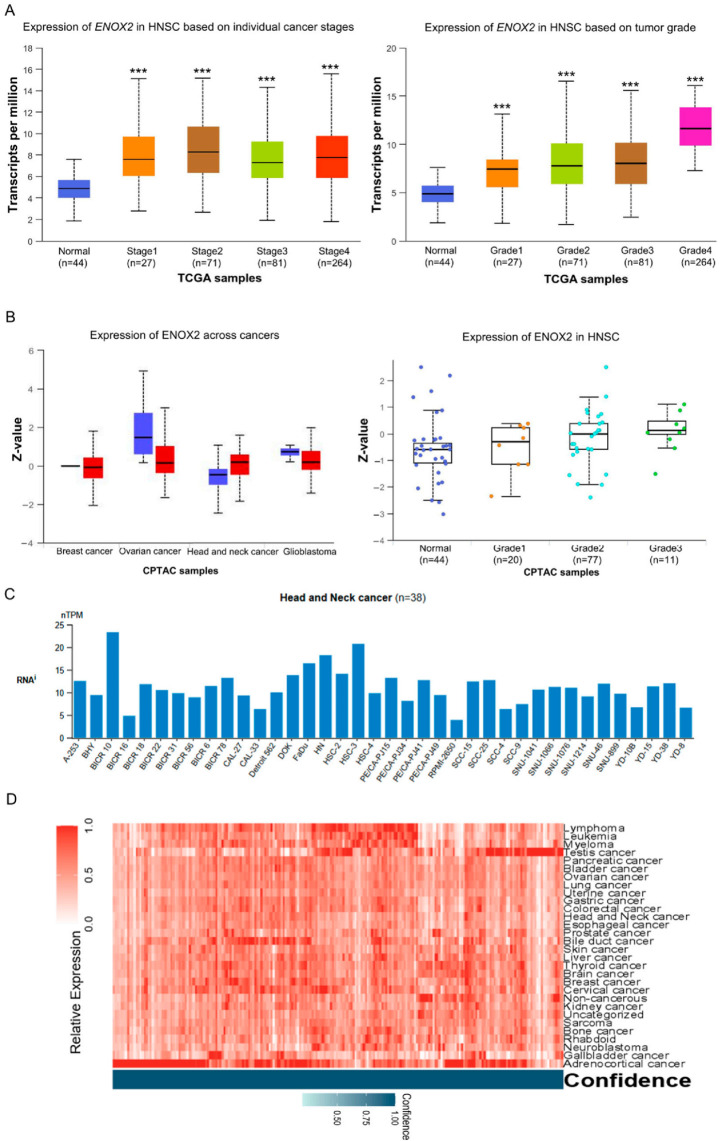
Expression of ENOX2 in head and neck cancer. (**A**,**B**). Differential expression of *ENOX2* in HNSCC and adjacent normal tissues, analyzed from the TCGA-HNSC (DOI: 10.1038/nature14129) (**A**) and CPTAC (**B**) datasets using UALCAN (https://ualcan.path.uab.edu). *ENOX2* mRNA levels were significantly higher in TCGA-HNSC tumor tissues than in normal tissues (*** *p* < 0.001), whereas CPTAC proteomic data showed a non-significant trend toward higher ENOX2 protein expression in tumors. (**C**). Analysis of transcriptomic data from the Human Protein Atlas (HPA) based on the CCLE dataset, showing that *ENOX2* mRNA expression varies among head and neck cancer cell lines, with relatively higher expression observed in the HSC-3 cell line. Statistical testing (*p*-values) was not available from the dataset. (**D**) Heatmap visualization of *ENOX2* mRNA expression across multiple cancer types in the HPA dataset, showing that *ENOX2* overexpression is not limited to HNSCC. Values are normalized within the dataset and are not directly compared to the housekeeping gene.

Oral cancer is associated with high morbidity and mortality worldwide. Previous studies showed that ENOX2 contributes to cancer progression by regulating SIRT1, thereby implicating it in tumor development [31,49,50]. However, the expression of ENOX2 in oral cancer tissues relative to normal tissues has not been fully characterized. To address this, we conducted in silico analyses using publicly available databases to assess ENOX2 expression in patient-derived oral cavity tumors. Databases providing high-throughput mRNA and protein data, including UALCAN, cBioPortal, and the Human Protein Atlas, were utilized to compare *ENOX2* expression in tumor tissues versus matched normal counterparts. Analysis of The Cancer Genome Atlas (TCGA) Pan-Cancer dataset revealed that *ENOX2* transcript expression was significantly elevated in head and neck squamous cell carcinoma (HNSCC) tissues compared with normal tissues, with correlations seen across different tumor stages and grades (Figure 2A). Similarly, data from the Clinical Proteomic Tumor Analysis Consortium (CPTAC) indicated that ENOX2 protein levels were higher in HNSCC tumor tissues than in normal tissues, with observable but non-significant trends for correlation seen across tumor grades (Figure 2B). Consistent with these findings, data from the Human Protein Atlas (https://www.proteinatlas.org) showed that ENOX2 mRNA expression was elevated in head and neck cancer cell lines, with relatively higher levels observed in HSC-3 cells (Figure 2C). Furthermore, heatmap analysis of transcriptomic data from the same database demonstrated that *ENOX2* mRNA expression was elevated across multiple cancer types, including head and neck cancer (Figure 2D). Collectively, these findings suggest that ENOX2 is upregulated in HNSCC and may play a role in tumor development and progression.

**Figure 3:**
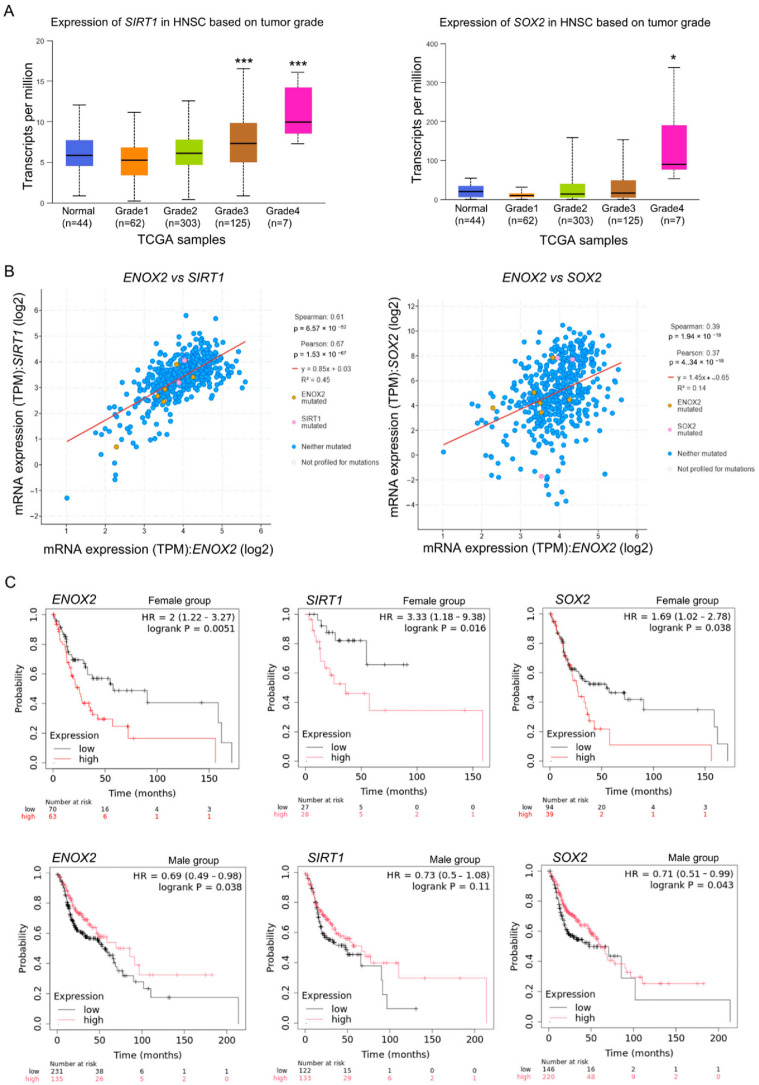
Expression and clinical relevance of *ENOX2, SIRT1*, and *SOX2* in head and neck cancer datasets. (**A**) TCGA analysis revealed significantly elevated *SIRT1* and *SOX2* transcript levels in HNSCC tumor tissues compared to normal tissues across all histological grades (* *p* < 0.05, *** *p* < 0.001). (**B**) Pearson correlation analysis demonstrated positive correlations between *ENOX2* and *SIRT1*, as well as between *ENOX2* and *SOX2*. (**C**) Kaplan–Meier plotter analysis indicated that high mRNA expression levels of *ENOX2* and *SOX2* were significantly associated with poor overall survival in both female and male patients with head and neck cancer, whereas *SIRT1* expression showed a significant association only in the female group (stage III + IV).

Similar to the results obtained for *ENOX2*, the transcript levels of *SIRT1* and *SOX2* were also elevated in HNSCC tumor tissues compared with normal tissues across different tumor grades (Figure 3A). Sex-determining region Y box 2 (*SOX2*) is a well-established transcription factor involved in stem cell maintenance and has been implicated in HNSCC stemness regulation [51,52]. Pearson correlation analysis of the TCGA-HNSC dataset revealed positive correlations between *ENOX2* and *SIRT1*, as well as between *ENOX2* and *SOX2* (Figure 3B). Because the TCGA-HNSC cohort includes tumors with diverse mutational profiles—particularly frequent *TP53* alterations—these associations likely reflect both transcriptional co-regulation and mutation-dependent expression patterns. Kaplan–Meier survival analysis using the RNA-seq dataset from the Kaplan–Meier Plotter (https://www.kmplot.com/) showed that high mRNA expression levels of *ENOX2* and *SOX2* were significantly associated with poor overall survival in both male and female patients, whereas *SIRT1* expression showed a significant correlation only in the female group (stage III + IV) (Figure 3C). Collectively, these findings suggest that *ENOX2*-associated signaling may contribute to stemness and tumor progression in head and neck cancer, potentially in a sex- and mutation-dependent manner.

### 3.2. ENOX2 Stimulates Stemness and Cell Proliferation in Oral Cancer Cells

**Figure 4:**
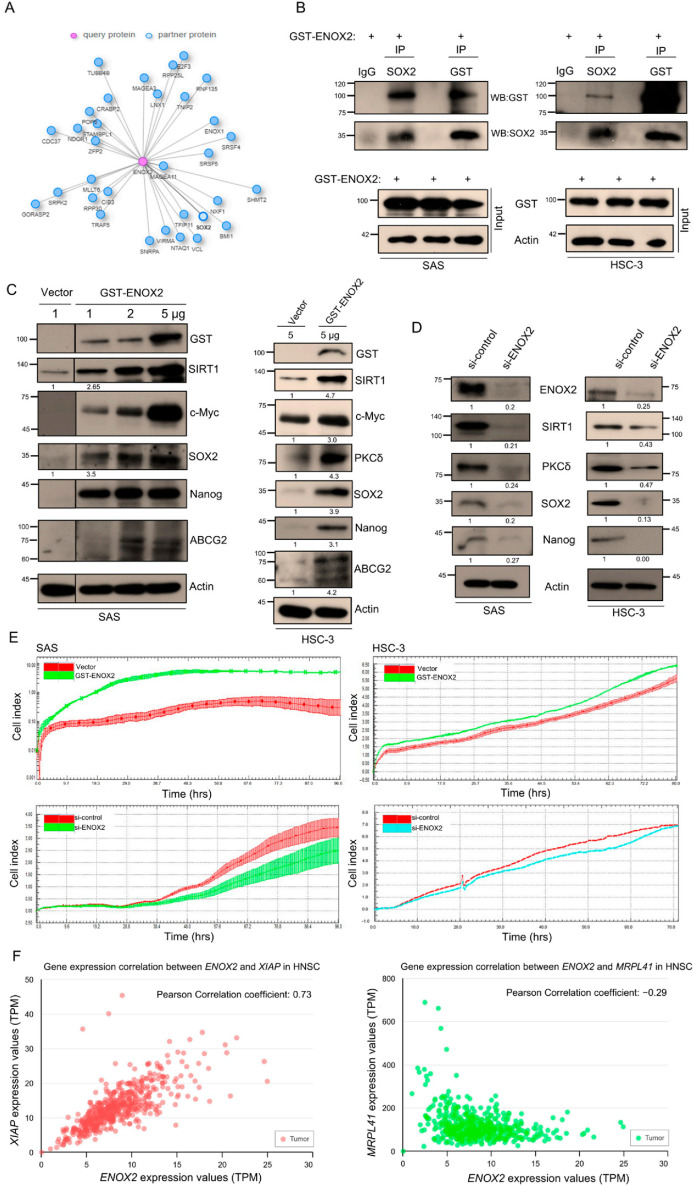
ENOX2 prompts stem-like properties and proliferation in oral cancer cells. (**A**) Interaction network of ENOX2 and associated proteins generated using the Integrated Interaction Database (IID). (**B**) Co-immunoprecipitation of GST-ENOX2-overexpressing cell lysates with non-immune IgG or antibodies against GST and SOX2, followed by Western blotting with anti-GST or anti-SOX2 antibodies. Whole-cell lysates were also immunoblotted with anti-ENOX2 and anti-SOX2 antibodies; β-actin served as a loading control. Representative images are shown. (**C**,**D**) Western blot analysis of stemness markers in cells transfected with GST or GST-ENOX2 for 72 h (**C**), or with si-control (scramble RNAi) or si-ENOX2 for 48 h (**D**). β-actin was used as the loading control. (**E**) Real-time monitoring of proliferation in SAS and HSC-3 cells transfected with GST, GST-ENOX2, si-control, or si-ENOX2. After overnight attachment, cells were seeded onto E-plates, and proliferation was continuously measured using the xCELLigence system. Cell index values are presented. (**F**) Pearson correlation analysis of ENOX2 with XIAP and MRPL41 in TCGA tumor tissues using UALCAN (https://ualcan.path.uab.edu).

Given the positive correlation between ENOX2 and SOX2, we investigated the relationship between these two proteins in oral cancer. SOX2 is a transcription factor that is crucial for maintaining the self-renewal and pluripotency of embryonic stem cells, while ENOX2 is a growth-related protein that contributes to cancer regulation [32,53]. Analysis using the Integrated Interaction Database (IID) (https://iid.ophid.utoronto.ca/search_by_proteins/ (accessed on 25 June 2024)) suggested a potential functional association between ENOX2 and SOX2 (Figure 4A). To test whether ENOX2 physically interacts with SOX2, we performed a co-immunoprecipitation assay. Our results showed that ENOX2 co-precipitated with SOX2 in SAS (wild-type *p53*) oral cancer cells (Figure 4B). Because SOX2 is implicated in HNSCC stemness regulation, we next investigated whether ENOX2 influences stemness-related characteristics in these oral cancer cell lines. Overexpression of ENOX2 markedly increased the expression levels of stemness markers, including SIRT1, c-Myc, SOX2, Nanog, and ABCG2, in both SAS and HSC-3 cells (Figure 4C). Conversely, ENOX2 knockdown reduced the expression of SIRT1, SOX2, and Nanog (Figure 4D). These results suggest that ENOX2 may contribute to maintaining stemness in oral cancer cells, potentially through indirect modulation of SIRT1-SOX2 signaling.

Consistent with previous reports in cancer models [54,55,56], ENOX2 overexpression increased SIRT1 levels, whereas ENOX2 knockdown decreased SIRT1 expression. We also found that the expression of PKCδ, a serine/threonine kinase of the PKC family, was modulated by both ENOX2 overexpression (Figure 4C) and knockdown (Figure 4D). Given that ENOX2 can be phosphorylated by PKCδ and thereby modulate cell proliferation and migration [57], the regulatory hierarchy between ENOX2 and PKCδ requires further clarification. In line with previous studies showing that ENOX2 regulates cancer cell growth and migration [32,48,57], we found that ENOX2 overexpression promoted cell proliferation, whereas ENOX2 depletion reversed this effect in both SAS and HSC-3 cells (Figure 4E). Because ENOX2 is also linked to cell death regulation [31,49,50,58], we next explored genes correlated with ENOX2 in HNSCC datasets. Notably, XIAP, an inhibitor of apoptosis, was positively correlated with ENOX2 expression, whereas MRPL41, an apoptosis inducer, was negatively correlated (Figure 4F). While these findings are consistent with a role for ENOX2 in balancing pro-and anti-apoptotic pathways, the causal mechanisms remain to be established.

### 3.3. Sphere Formation and Stemness Characteristics in p-53 Functional SAS and p-53 Mutated HSC-3 Cells

**Figure 5:**
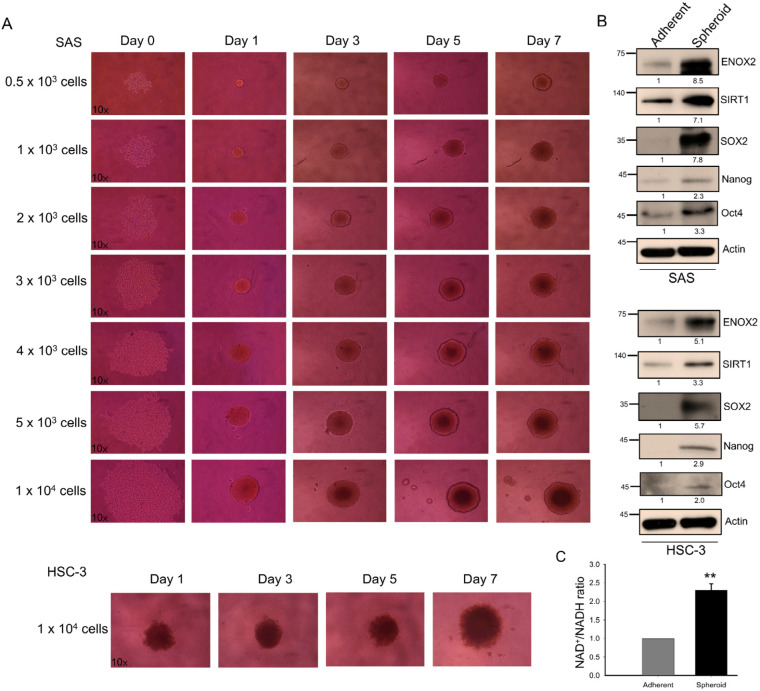
Sphere formation and stem-like properties of SAS and HSC-3 oral cancer cells. (**A**) Sphere-forming abilities of SAS and HSC-3 cells. Various cell densities of SAS cells (0.5 × 10^3^, 1 × 10^3^, 2 × 10^3^, 3 × 10^3^, 4 × 10^3^, 5 × 10^3^, and 1 × 10^4^) and HSC-3 cells (1 × 10^4^) were seeded into 96-well ultra-low attachment plates and cultured in DMEM supplemented with 10% FBS at 37 °C. Representative images (magnification 10×) obtained at different time points are shown. (**B**) Western blot analysis of spheroid cells harvested at day 7, showing expression of ENOX2, SIRT1, and stemness-associated markers. β-Actin served as the loading control. (**C**) Comparison of the intracellular NAD^+^/NADH ratios between adherent and spheroid SAS cells, as measured using an NADH/NAD quantification kit. Data are presented as mean ± SD from three independent experiments (** *p* < 0.01).

Sphere formation, the aggregation of cells into 3D structures under non-adherent conditions, is an in vitro method that is widely used to assess stem-like properties. Consistent with a previous report [59], both *p53*-functional SAS and *p53*-mutant HSC-3 oral cancer cell lines formed spheres at varying seeding densities in our system (Figure 5A). Compared with adherent monolayer cultures, spheroid cells exhibited elevated levels of expression of ENOX2, SIRT1, and the stemness-associated markers, SOX2, Nanog, and Oct4 (Figure 5B). Notably, both the protein level and enzymatic activity of ENOX2, as reflected by the NAD^+^/NADH ratio, were markedly higher in spheroids than in adherent cells (Figure 5B,C), supporting the idea that the spheroids were enriched with a stem-like subpopulation.

**Figure 6:**
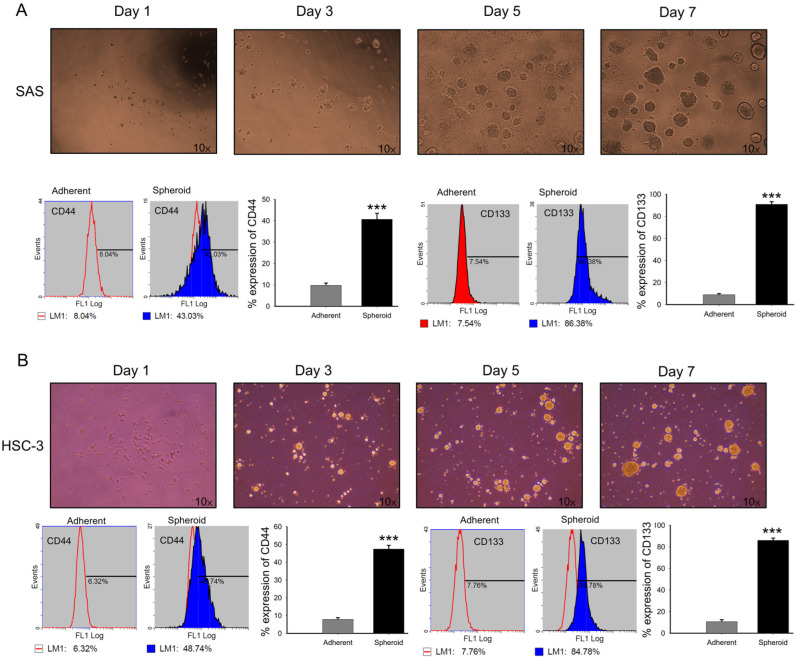
Expression of CD44 and CD138 in SAS and HSC-3 spheroid cells. (**A**,**B**) SAS and HSC-3 cells were cultured in poly-HEMA-coated 10 cm dishes under non-adherent conditions. After 7 days, spheroids were dissociated into single cells stained with FITC-conjugated antibodies against CD44 and CD138, and analyzed by flow cytometry. Percentages of positive cells were determined. Both markers were significantly increased in spheroid cultures compared with adherent controls. Data are presented as mean ± SD from at least three independent experiments (*** *p* < 0.001 vs. control).

Under non-adherent, serum-free conditions, spheroid cultures provide a selective microenvironment that favors the survival and expansion of cells with stem-like characteristics [60]. Within these cultures, a subpopulation of cancer cells with stem-like features emerges, often defined by increased expression of specific surface markers. To validate this, 7-day SAS (Figure 6A) and HSC-3 (Figure 6B) spheroids were dissociated into single cells and analyzed by flow cytometry for CD44, a well-recognized CSC marker, and CD138 (syndecan-1), which is associated with epithelial and tumorigenic properties. Both markers were significantly upregulated in spheroid cultures compared with adherent cells (Figure 6), supporting the enrichment of a stem-like subpopulation.

### 3.4. ENOX2 Regulates Sphere Formation and Stemness in Oral Cancer Cells

**Figure 7:**
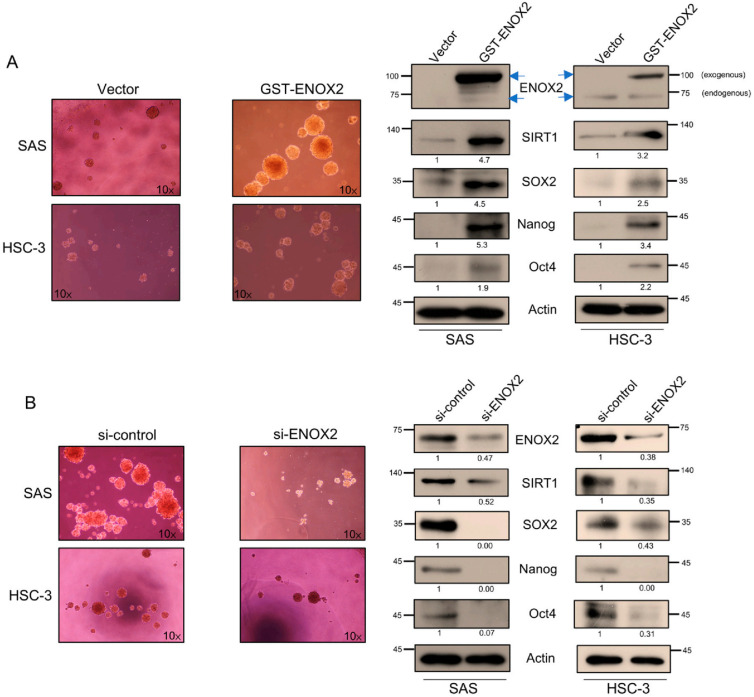
Role of ENOX2 in regulating the stemness of oral cancer cells. (**A**) SAS and HSC-3 cells were transfected with GST or GST-ENOX2 for 72 h, seeded onto poly-HEMA-coated 6-well plates, and cultured under non-adherent conditions. After 7 days, spheroids were harvested, and protein levels of stemness-associated markers were analyzed by Western blotting. (**B**) SAS and HSC-3 cells were transfected with si-control or si-ENOX2 for 48 h, seeded onto poly-HEMA-coated 6-well plates, and cultured under non-adherent conditions. After 7 days, spheroids were harvested and analyzed by Western blotting. β-Actin was used as the loading control.

We observed that both ENOX2 and SIRT1 were upregulated in spheroid cultures, a system that selectively enriches for cancer stem-like cells. This suggests that their elevated expression is likely functionally associated with sustaining CSC properties, such as self-renewal and survival, rather than being a downstream byproduct of spheroid growth or the initiation of stemness traits. To further explore the role of ENOX2 in stem-like cancer cell populations, we transiently overexpressed ENOX2 in two oral cancer cell lines: SAS cells (*p53*-functional) and HSC-3 cells (*p53*-mutant). ENOX2 overexpression was found to significantly enhance sphere formation, a hallmark of CSC self-renewal (Figure 7A, left panel), and increased the expression of SIRT1 and stemness-associated markers, SOX2, Nanog, and Oct4 (Figure 7A, right panel). Conversely, RNAi-mediated knockdown of ENOX2 reduced sphere formation (Figure 7B, left panel) and downregulated SIRT1, SOX2, Nanog, and Oct4 (Figure 7B, right panel). These molecular changes were accompanied by signs of differentiation, supporting the hypothesis that ENOX2 is likely required for maintenance of stem-like traits. Collectively, our findings demonstrate that ENOX2 promotes CSC-like properties in oral cancer cells, at least in part through regulation of SIRT1 and stemness-associated transcription factors. However, loss of ENOX2 disrupts these pathways, potentially driving cells toward differentiation, highlighting ENOX2 as a potential therapeutic target in oral cancer.

### 3.5. ENOX2 Drives Oral Cancer Stem Cell Formation In Vivo

**Figure 8:**
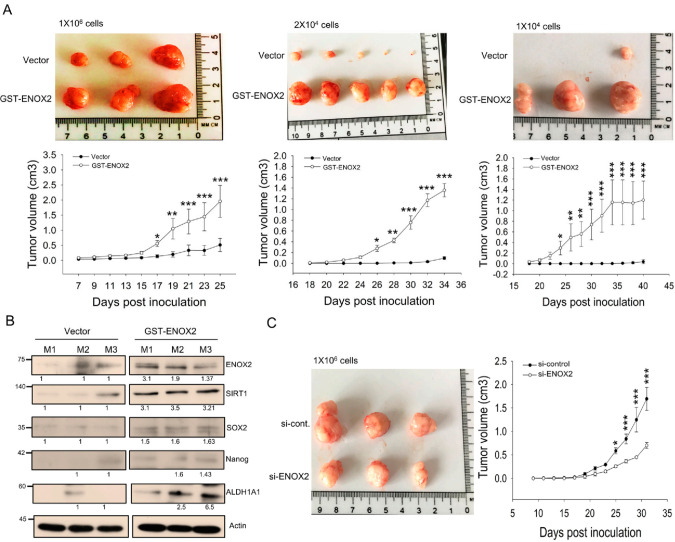
ENOX2 promotes the growth of oral cancer xenografts in ASID mice. (**A**) SAS cells were transiently transfected with GST or GST-ENOX2. After 72 h, cells were cultured in poly-HEMA–coated plates to form spheres. Following 7 days of sphere formation, spheroids were dissociated into single cells and subcutaneously inoculated into ASID mice at doses of 1 × 10^6^, 2 × 10^4^, or 1 × 10^4^. Tumor growth was assessed by measuring tumor volume. Representative tumor morphology and quantitative analyses are shown. Statistically significant differences between the GST and GST-ENOX2 groups were determined by one-way ANOVA with LSD post hoc test (* *p* < 0.05, ** *p* < 0.01, *** *p* < 0.001). (**B**) Tumor tissues from both groups were homogenized and analyzed by Western blotting for stemness-associated markers. (**C**) Sphere-forming cells transfected with si-control or si-ENOX2 (1 × 10^6^) were subcutaneously inoculated into ASID mice (n = 3 per group). Tumor growth, measured by assessment of tumor volume, was significantly reduced in the si-ENOX2 group compared with controls (* *p* < 0.05, *** *p* < 0.001).

Stem cells are increasingly being recognized as drivers of tumor development and progression. In a previous study, we showed that ENOX2 knockdown in melanoma cells significantly inhibited tumor formation [33]. Building on this, we herein demonstrated that ENOX2 overexpression promotes stem-like properties, whereas ENOX2 silencing suppresses stemness in oral cancer cells in vitro. To assess the role of ENOX2 in vivo, we employed xenograft mouse models. Spheroid-derived oral cancer cells transiently overexpressing ENOX2 exhibited markedly enhanced tumor-initiating capacity compared with vehicle control cells (Figure 8A). Consistent with this increased tumor growth, tumors derived from ENOX2-overexpressing cells expressed significantly higher levels of stemness-associated markers, as determined by Western blot analysis (Figure 8B). Conversely, mice inoculated with ENOX2-knockdown spheroid cells displayed substantially smaller tumor volumes relative to controls (Figure 8C). Collectively, our findings indicate that ENOX2 enhances tumor initiation and stemness in oral cancer cells, while its depletion impairs these properties. These results support the notion that ENOX2 plays a critical role in maintaining cancer stem cell characteristics and promoting tumor progression in vivo.

### 3.6. The ENOX2 Signaling Modulator, Capsaicin, Inhibits Sphere Formation and Downregulates Stemness Marker Expression in Oral Cancer Cells In Vitro

**Figure 9:**
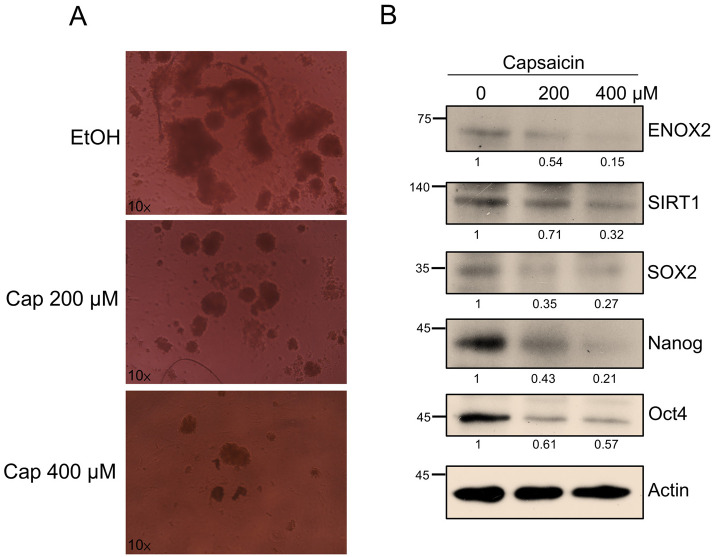
Capsaicin suppresses sphere formation and stemness marker expression in SAS cells. (**A**) SAS cells were cultured in ultra-low attachment plates for 48 h, and then treated with or without capsaicin for 5 days. Representative images of spheres at day 7 are shown. (**B**) Western blot analysis of SAS spheroids treated with or without capsaicin. Protein levels of ENOX2, SIRT1, and stemness-associated markers (SOX2, Nanog, Oct4) were assessed. β-Actin served as a loading control.

Having established that ENOX2 promotes oral cancer stemness and tumor growth both in vitro and in vivo, we next explored whether targeting this pathway could represent a therapeutic strategy. Capsaicin, a dietary bioactive compound from chili peppers, has been reported to exert anti-cancer effects, including inhibition of proliferation and induction of apoptosis through modulation of ENOX2 activity and related redox signaling [33]. Beyond oncology, capsaicin also influences stem cell behavior [61]. For example, it reportedly inhibits the differentiation of bone marrow stem cells (BMSCs) into adipocytes by inducing apoptosis, suppressing adipocyte-specific gene expression, and reducing BMSC proliferation [62]. In this study, we investigated whether capsaicin could affect oral cancer stem-like properties via interference with the ENOX2–associated pathways. Treatments with capsaicin (200 µM and 400 µM) significantly reduced sphere formation in SAS cells (Figure 9A). Western blot analysis further revealed that capsaicin suppressed the expression levels of ENOX2, SIRT1, and the stemness-associated markers, SOX2, Nanog, and Oct4 (Figure 9B). These results suggest that capsaicin may modulate the ENOX2-associated redox pathway, leading to altered NAD^+^-dependent SIRT1 signaling and a subsequent decrease in stemness-related transcriptional activity. We therefore propose a hypothetical model in which capsaicin indirectly influences the ENOX2–SIRT1–SOX2 regulatory network, resulting in attenuation of cancer stem-like properties. Further biochemical and mechanistic studies are needed to confirm whether this interaction is direct or mediated through secondary pathways.

## 4. Discussion

Oral cancer remains a major health concern, particularly in Taiwan, where a nationwide screening program has been implemented. However, recurrence and metastasis continue to pose formidable challenges [3,4]. These clinical realities highlight the need for therapeutic approaches that move beyond early detection to directly target the mechanisms of relapse. The recognition of cancer stem cells (CSCs) has provided critical insight into this problem, as their intrinsic capacity for self-renewal and pluripotency underlies tumor persistence, therapy resistance, and disease recurrence [63,64]. Among the transcriptional regulators that orchestrate stemness, SOX2 plays a central role within a network of pluripotency factors (e.g., Oct4 and Nanog) to sustain an undifferentiated state and govern cell fate [65,66,67,68]. Our present findings suggest that ENOX2 may act as an upstream regulator of SOX2 in oral cancer cells. Gain-of-function experiments demonstrated that ENOX2 promotes a stem-like phenotype by enhancing SOX2 expression, likely in part through the modulation of NAD^+^-dependent SIRT1 activity, a protein deacetylase known to regulate both embryonic and somatic stem cell function [69,70]. Importantly, SIRT1 is a versatile regulator of SOX2, influencing its stability through both post-translational and transcriptional mechanisms to sustain stemness [39,71]. Consistent with these observations, ENOX2 knockdown reduced SIRT1, SOX2, and pluripotency markers while promoting signs of differentiation, supporting the hypothesis that ENOX2 contributes to CSC maintenance.

In vivo, ENOX2 overexpression increased the expression levels of SIRT1 and SOX2 in tumors, further reinforcing the notion that ENOX2 contributes to maintaining CSC-like traits. The proposed ENOX2-SIRT1 regulatory relationship aligns with prior studies implicating this axis in proliferation, migration, epithelial–mesenchymal transition (EMT), apoptosis, and autophagy [33,49,56,72]. Collectively, our results suggest that ENOX2 may function within a broader signaling framework that regulates stemness and tumor progression, rather than as a single linear pathway. The mechanistic role of PKCδ in phosphorylating ENOX2 and regulating its activity, as previously reported [57], further supports the concept that ENOX2 acts as a multifunctional node integrating redox, signaling, and growth control mechanisms. This complexity underscores the need for comprehensive mapping of ENOX2’s interacting partners and post-translational modifications to better define its contribution to CSC biology.

Our mechanistic observations also gain translational relevance in the context of capsaicin, a dietary bioactive compound with documented anti-cancer properties [73,74,75,76,77,78]. Previous studies have shown that capsaicin directly interacts with ENOX2, promoting its degradation through both the ubiquitin–proteasome and autophagy–lysosome pathways, which in turn inhibits SIRT1 activity and induces cancer cell death [33,58]. In our study, capsaicin reduced ENOX2, SIRT1, and stemness-associated markers, suggesting its potential role in modulating this regulatory network. However, we emphasize that this represents a proposed model based on correlative evidence and does not imply direct binding or therapeutic efficacy. Further biochemical and pharmacological studies will be required to elucidate whether capsaicin interacts directly with ENOX2 or acts through secondary redox-regulated pathways.

This study has several limitations. First, while multiple in vitro and in vivo models were used, the mechanistic interactions within the ENOX2–SIRT1–SOX2 axis remain partially hypothetical and require further validation through rescue assays and signaling analyses. Second, our bioinformatic analyses relied on publicly available datasets, which may be influenced by sample heterogeneity and limited annotation of oral-specific subtypes. Third, although capsaicin’s inhibitory effects on ENOX2-associated stemness are consistent with prior literature, additional biochemical assays—such as enzyme activity inhibition or co-crystallization studies—are needed to confirm direct interactions. Future work should integrate patient-derived organoids, clinical datasets, and proteomic profiling to establish the translational potential of targeting ENOX2 in oral cancer.

Taken together, our findings highlight ENOX2 as a potential regulator of CSC-like traits in oral cancer, acting through a putative SIRT1–SOX2–mediated mechanism. While the proposed ENOX2–SIRT1–SOX2 framework provides a valuable conceptual basis, further mechanistic and translational studies are needed to confirm its functional and therapeutic relevance.

## 5. Conclusions

In summary, this study provides evidence that ENOX2 contributes to the maintenance of stem-like characteristics in oral cancer cells, potentially through modulation of the SIRT1–SOX2 axis. Using integrated in vitro, in vivo, and bioinformatic analyses, we propose a mechanistic framework in which ENOX2-associated redox activity influences NAD^+^-dependent signaling and stemness regulation. Although the current findings support a connection between ENOX2, SIRT1, and SOX2, the proposed ENOX2–SIRT1–SOX2 network remains hypothetical and requires further mechanistic validation. Future studies should focus on dissecting the molecular basis of this interaction and assessing its translational potential as a therapeutic target in oral cancer.

## Data Availability

The original contributions presented in this study are included in the article/Supplementary Materials. Further inquiries can be directed at the corresponding author.

## Supplementary Materials

The following supporting information can be downloaded at: https://www.mdpi.com/article/10.3390/antiox15010098/s1, Supplementary Table S1: provides a list of all antibodies and reagents used in this study, including their respective vendors, catalog numbers, and working dilutions. The uncropped original Western blot images, together with the quantitative analyses and associated statistical data (bar graphs), are presented in the Supplementary Figures.

## Abbreviations

The following abbreviations are used in this manuscript:

2D	two-dimensional
3D	three-dimensional
CSCs	Cancer stem cells
ENOX2, tNOX	Tumor-associated NADH oxidase
Sirtuin 1, SIRT1	Silent information regulator 1
SOX2	Sex-determining region Y box 2
TCGA	The Cancer Genome Atlas
HNSCC	Neck squamous cell carcinoma
CPTAC	Clinical Proteomic Tumor Analysis Consortium
