# Supplementary material for: ENOX2 (tNOX)–Associated Stemness in Oral Cancer Cells and Its Clinical Correlation in Head and Neck Tumors
Source: Antioxidants (Basel). 2026 Jan 13;15(1):98. doi: 10.3390/antiox15010098 (PMC12837915; doi:10.3390/antiox15010098)
Supplement: Supplementary file 1 [file antioxidants-15-00098-s001.zip › antioxidants-3920581-Supplementary-Table.pdf]

Supplementary Table S1. Antibodies and reagents used in this study

Antibodies/Reagents	Company	Catalog numbers	Dilution
SIRT1	Cell Signaling Technology, Danvers, MA, USA	2496	1:1000
Nanog	Cell Signaling Technology, Danvers, MA, USA	4903	1:2000
SOX2	Cell Signaling Technology, Danvers, MA, USA	2748	1:1000
ALDH1A1	Cell Signaling Technology, Danvers, MA, USA	54135	1:1000
ABCG2	Cell Signaling Technology, Danvers, MA, USA	4477	1:1000
Oct4	Cell Signaling Technology, Danvers, MA, USA	2750	1:1000
PKCδ	Cell Signaling Technology, Danvers, MA, USA	2058	1:1000
c-Myc	Cell Signaling Technology, Danvers, MA, USA	5605	1:1000
GST	Cell Signaling Technology, Danvers, MA, USA	2624	1:1000
CD44-FITC	Invitrogen, Carlsbad, CA, USA	11-0441-82	1:50
Anti-mouse IgG	Jackson ImmunoResearch Laboratories, West Grove, PA, USA	115-035-003	1:20,000
Anti-rabbit IgG	Jackson ImmunoResearch Laboratories, West Grove, PA, USA	111-035-003	1:20,000
β-actin	Proteintech, Rosemont, IL, USA	60008–1-Ig	1:10,000
CD133 (Prominin-1)	Sigma Aldrich, Burlington, MA, USA	ZRB1013	1:50
Mouse anti-rabbit IgG-FITC	Sigma Aldrich, Burlington, MA, USA	AP160F	1:200

Capsaicin	Sigma Aldrich, Burlington, MA, USA	M2028	
Poly(2-hydroxyethyl methacrylate)	Sigma Aldrich, Burlington, MA, USA	25249-16-5	
