# Supplementary figures and images for: ENOX2 (tNOX)–Associated Stemness in Oral Cancer Cells and Its Clinical Correlation in Head and Neck Tumors
Source: Antioxidants (Basel). 2026 Jan 13;15(1):98. doi: 10.3390/antiox15010098 (PMC12837915; doi:10.3390/antiox15010098)

Figure 4C

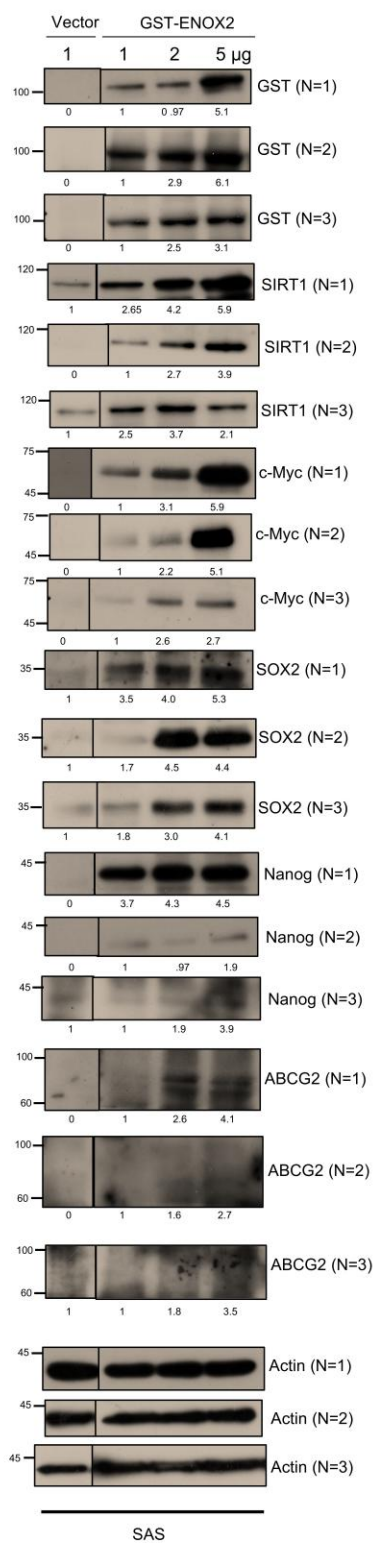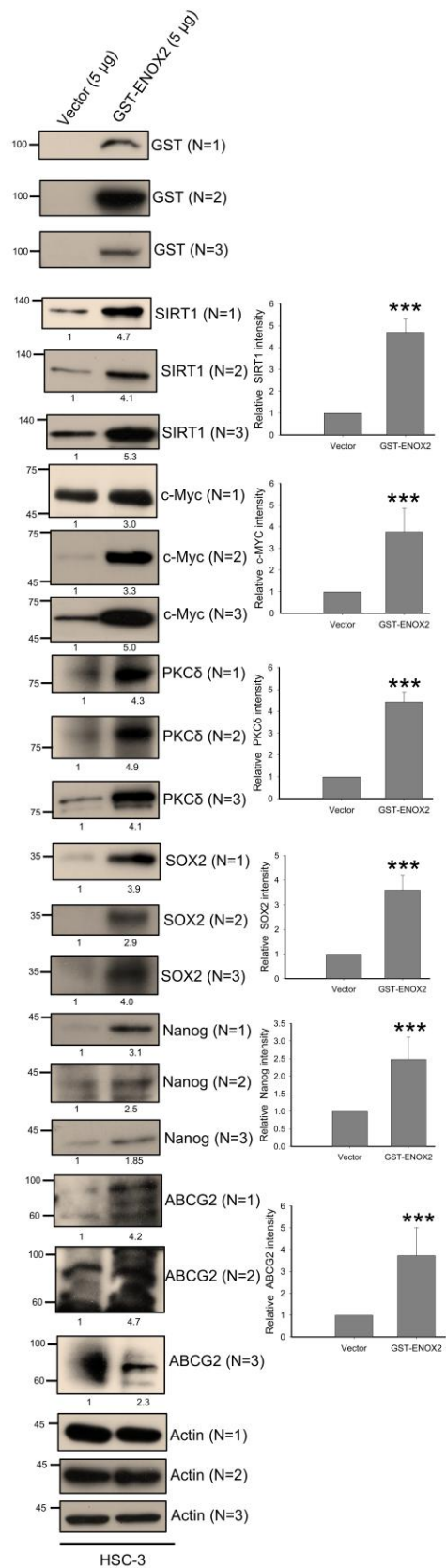

Figure 4D

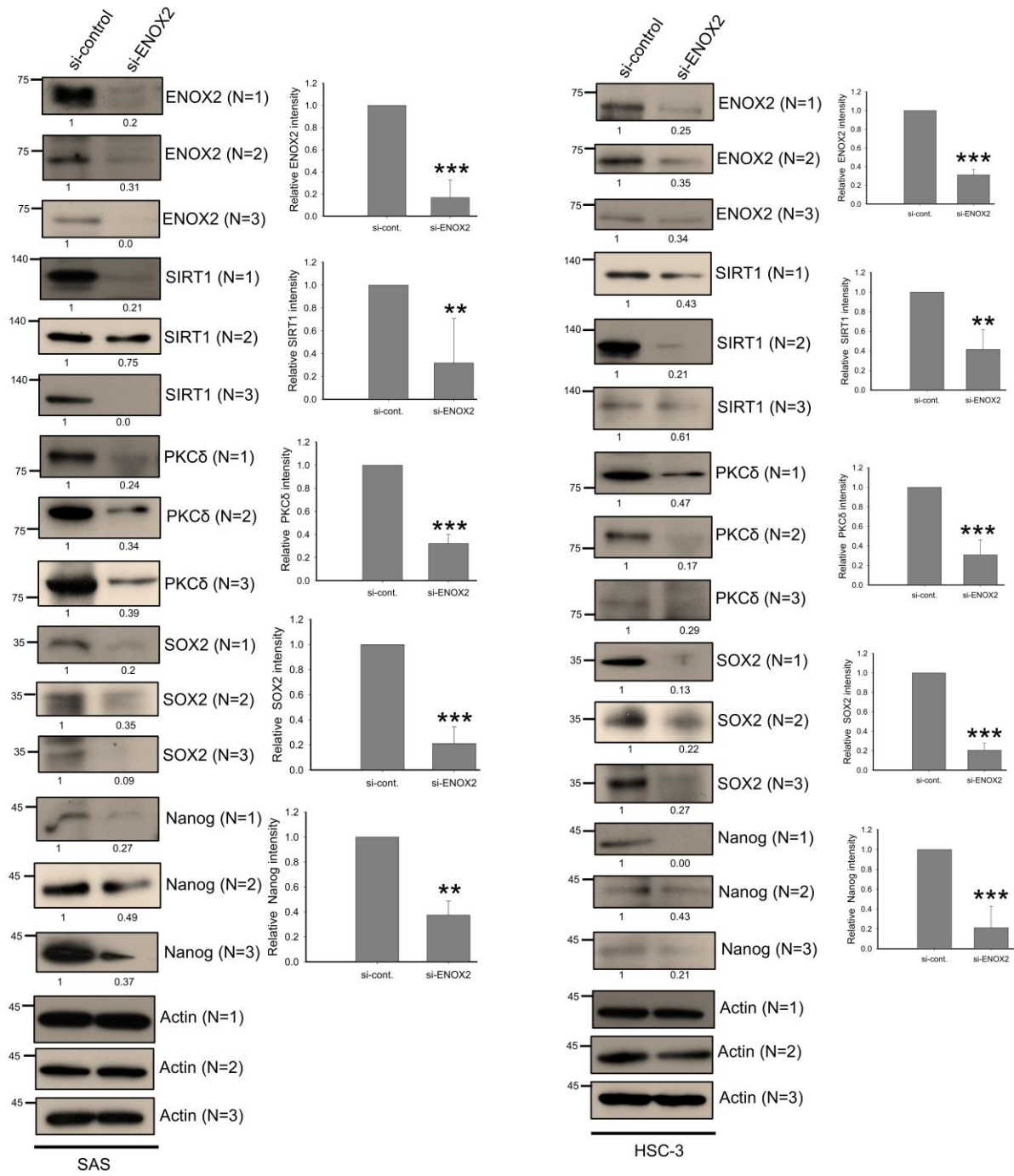

Figure 5B

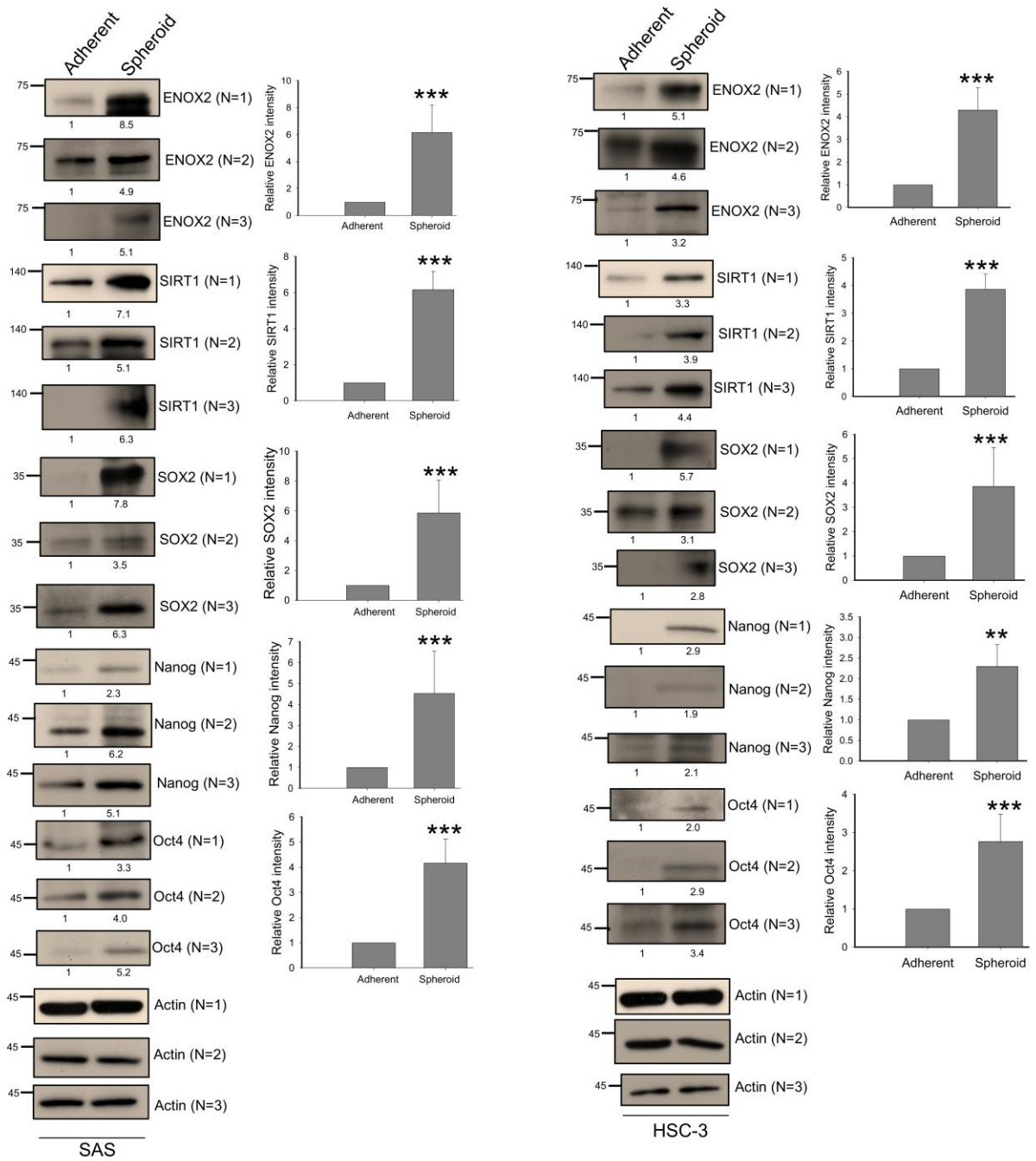

Figure 7A

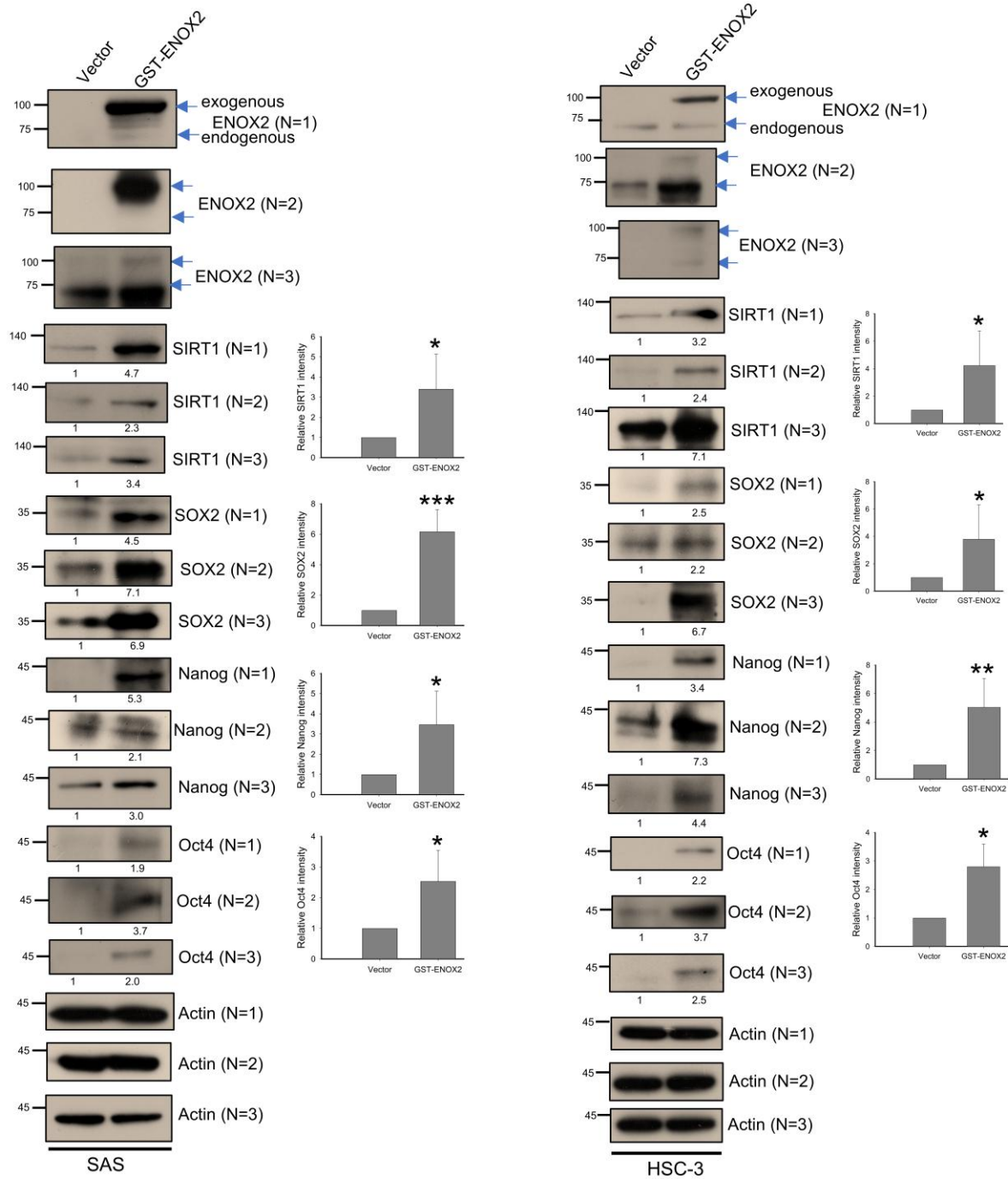

Figure 7B

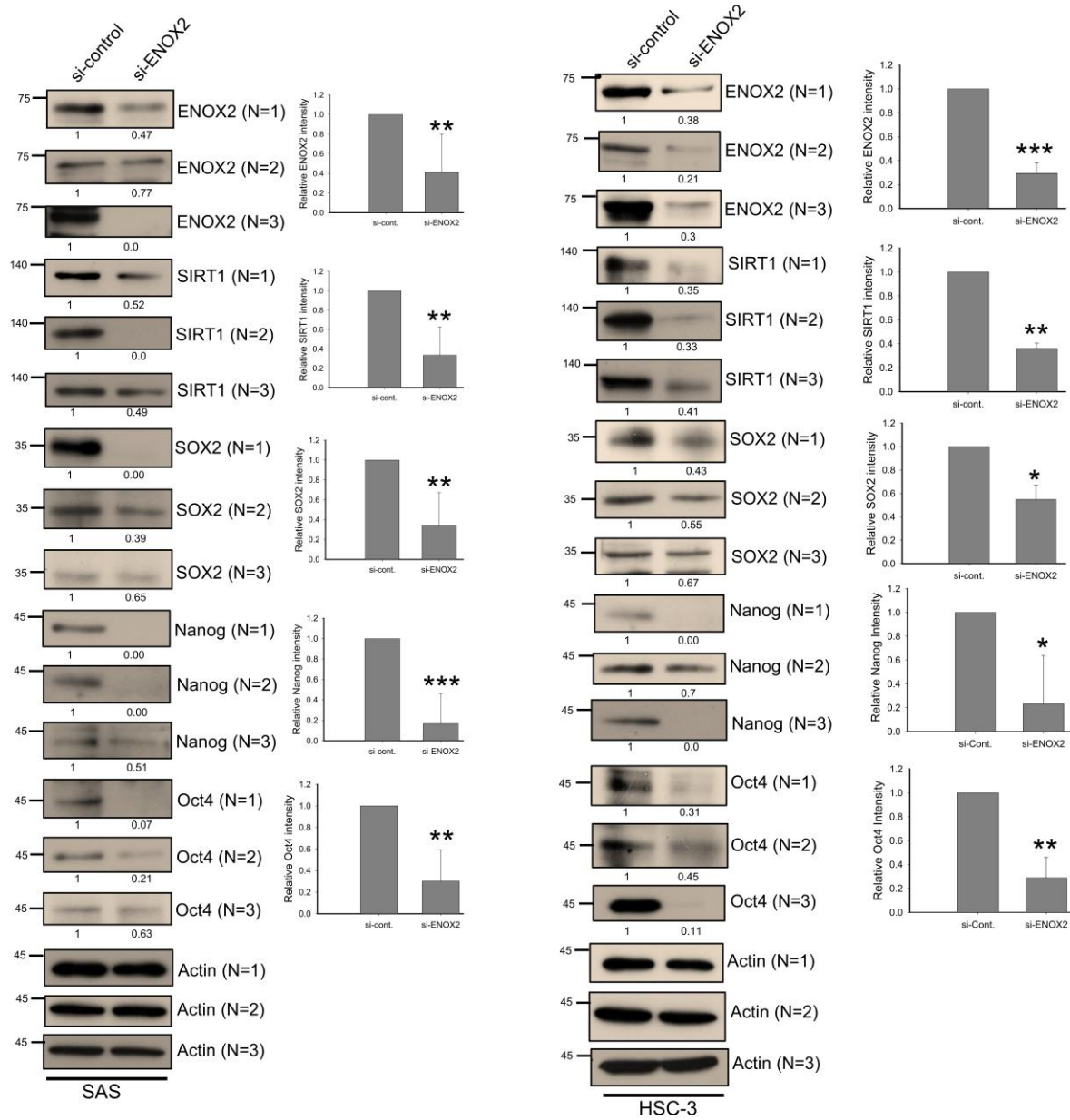

Figure 9B

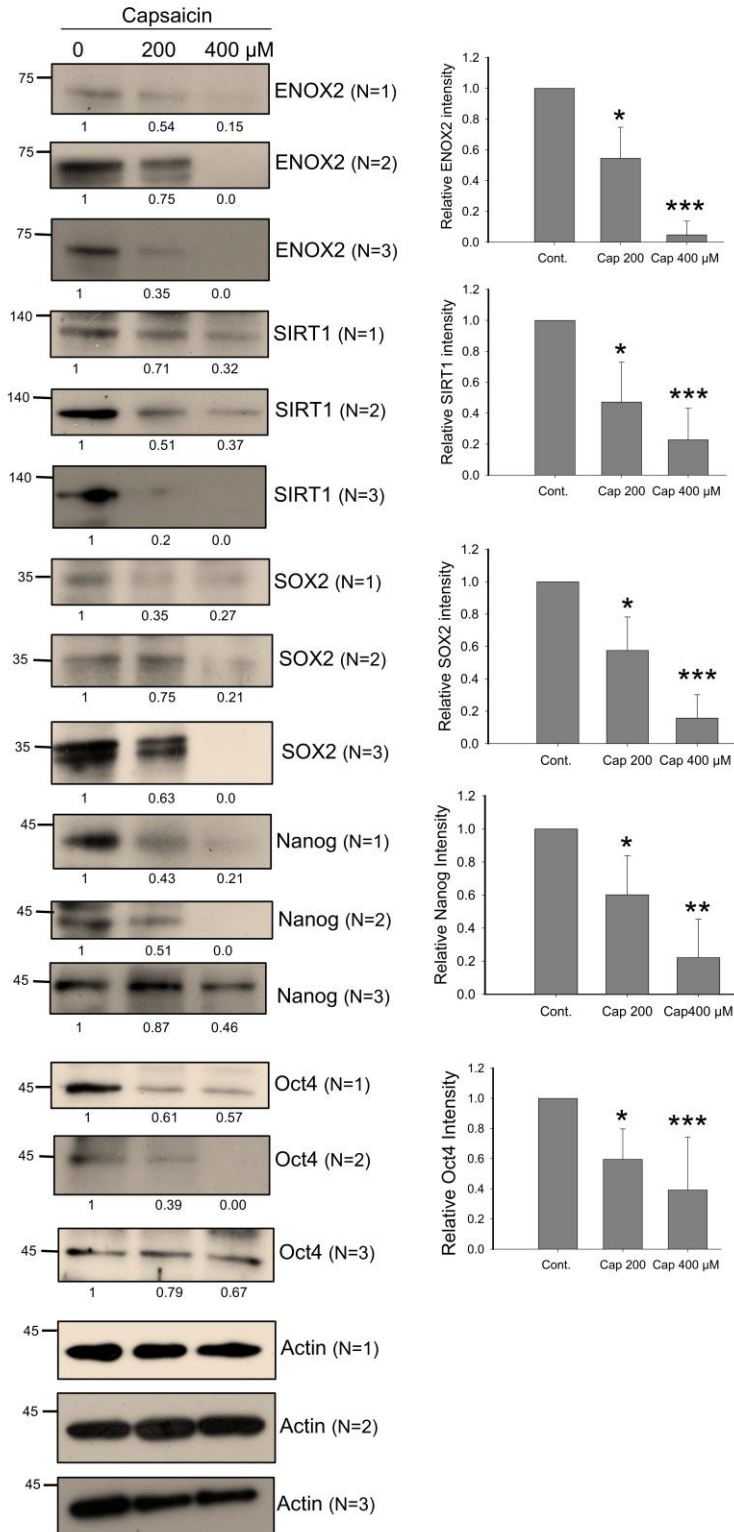

Supplement: Supplementary file 1 [file antioxidants-15-00098-s001.zip › antioxidants-3920581-Supplementary figure WB-1.6.2026.pdf]
